# Analysis of histology-agnostic targets among soft tissue and bone sarcomas in the AACR GENIE database

**DOI:** 10.3389/fonc.2022.1079909

**Published:** 2023-01-18

**Authors:** Roberto Carmagnani Pestana, César Serrano

**Affiliations:** ^1^ Hospital Israelita Albert Einstein, São Paulo, Brazil; ^2^ Department of Medical Oncology, Vall d’Hebron University Hospital, Barcelona, Spain; ^3^ Sarcoma Translational Research Program, Vall d’Hebron Institute of Oncology (VHIO), Hospital Universitario Vall d’Hebron, Vall d’Hebron Barcelona Hospital Campus, Barcelona, Spain

**Keywords:** sarcoma, histology-agnostic drug development, precision oncology, precision medicine, tissue-agnostic biomarker

## Abstract

**Background:**

The development of novel therapies for patients with sarcoma is challenging due to the rarity and diversity of these mesenchymal neoplasms. Hence, histology-agnostic approvals can be of particular interest for the treatment of patients with soft tissue and bone sarcoma.

**Methods:**

We queried the American Association for Cancer Research (AACR) Project Genomics Evidence Neoplasia Information Exchange (GENIE) database Cohort v12.0-Public to investigate the prevalence of currently Food and Drug Administration (FDA)-approved and other potentially actionable histology-agnostic alterations in patients with soft tissue and bone sarcoma. Targets were identified by a literature review by the authors. Results are presented for each cohort identified in the GENIE database, namely: (1) soft tissue sarcoma (STS), (2) gastrointestinal stromal tumor (GIST), (3) bone sarcoma, (4) uterine sarcoma, and (5) breast sarcoma.

**Results:**

We identified 7,512 samples of 6,955 patients with sarcoma in the AAACR GENIE database v12.0-Public. Molecular alterations that could lead to the clinical use of a currently approved histology-agnostic therapy were identified in 2.1% of sarcomas (2.6% STS, 1.3% GIST, 1.4% bone, 2.7% uterine, and 0% breast). In addition, 2.9% of patients could be eligible for future histology-agnostic approvals. These specific mutations, fusions, and amplifications occurred in multiple histotypes in all cohorts.

**Discussion:**

Exploring a public large-scale genomic database, we identified that 5% of patients with sarcoma could be eligible for current histology-agnostic FDA-approved drugs or future potential histology-agnostic indications. These actionable alterations were present in a wide variety of histologies in soft tissue and bone sarcomas, highlighting that next-generation sequencing can be considered for patients with advanced sarcoma to guide treatment strategies.

## 1 Background

Sarcomas are a heterogeneous group of rare cancers that share a mesenchymal origin. Specific subtypes of sarcomas, however, have distinct clinical, pathological, and molecular features, leading to disparate responses to the currently-approved standard of care therapies and variable overall prognosis (1). Still, regardless of such diverseness within sarcomas – the current World Health Organization (WHO) classification identifies approximately 100 histologic subtypes of sarcoma –, a one-size-fits-all approach has dominated the treatment of advanced soft tissue sarcoma (STS) in the past 40 years. Bone sarcomas have been managed similarly. Although significant advances in overall survival were initially achieved with chemotherapy, only anecdotal targeted or immune therapies have been approved for sarcomas. Therefore, there is an unmet clinical need to understand these tumors at the molecular level to “break the ceiling” and significantly impact the prognosis of these patients (2, 3).

The development of personalized, molecularly informed therapies is challenging in the diverse and rare group of sarcomas. Accordingly, only a small fraction of patients with soft tissue or bone sarcoma currently benefit from genome-targeted treatments (4–7). Scant biomarker-targeted therapies currently approved by the Food and Drug Administration (FDA) for sarcomas are those for KIT and PDGFRA in gastrointestinal stromal tumors (GIST), CSF1R in tenosynovial giant cell tumor, EZH2 in epithelioid sarcoma, mTOR in perivascular epithelioid cell differentiation tumors (PEComa), and ALK in inflammatory myofibroblastic tumor (4, 7–10).

In the past decade, the increasing understanding of molecular alterations responsible for carcinogenesis in multiple tumor types and the availability of highly active targeted therapies have ushered in a new era of drug development characterized by histology-agnostic, biomarker-driven therapies (11). In this new era, therapies are being developed to treat specific molecular alterations regardless of tumor tissue of origin. To date, the FDA has approved six drugs as histology-agnostic therapies, targeting four distinct molecular biomarkers (12–16).

Histology-agnostic development was first recognized as a novel regulatory pathway for drug approvals as a result of the identification of the microsatellite instability-high (MSI-H) phenotype as a predictive biomarker for the efficacy of anti-PD-1 immune-checkpoint inhibitors. This led to a series of trials investigating the use of pembrolizumab in patients with MSI-H tumors from various primary sources. The initial efficacy results were noticeable; the overall response rate (ORR) was 39% as an aggregate, including patients with 15 different tumor histologies. Moreover, the durability of such responses was impressive — 78% of responses were ongoing after six months (17). These results were the basis for the historical FDA histology-agnostic approval of pembrolizumab for patients with MSI-H tumors. Since then, the activity of pembrolizumab has been confirmed in a higher number of patients. In addition, another anti-PD-1 agent, dostarlimab-gxly, has been approved for the same indication (12, 14). Subsequently, larotrectinib and entrectinib were approved for solid tumors harboring an NTRK fusion, pembrolizumab was approved for solid tumors with high tumor mutational burden (TMB-H), and more recently, the combination of dabrafenib and trametinib was approved for solid tumors harboring a BRAF V600E mutation (13, 15, 16, 18). Moreover, therapies seeking such indications have expanded in recent years, beyond immune checkpoint inhibitors and targeted kinase inhibitors, to antibody-drug conjugates (19).

In the context of the difficulty of investigating specific targeted agents for the rare group of sarcomas, histology-agnostic drugs are of particular interest for the treatment of these mesenchymal malignancies, even though a limited number of patients with sarcoma were represented in the clinical trials leading to such approvals (20).

## 2 Objectives

The current analysis aims to describe the incidence and clinicopathologic correlates of histology-agnostic targetable alterations in soft tissue and bone sarcomas within the American Association for Cancer Research (AACR) Project Genomics Evidence Neoplasia Information Exchange (GENIE) database.

## 3 Material and methods

We queried the AACR GENIE database Cohort v12.0-Public to investigate the prevalence of currently approved and other potentially actionable histology-agnostic alterations in patients with soft tissue and bone sarcoma (21). Analyses were performed in accordance with the AACR GENIE Human Subjects Protection and Privacy policy.

First, we analyzed the targets with a current histology-agnostic approval by the FDA: namely, NTRK fusion, BRAF V600E mutation, MSI-H phenotype, and RET fusions. Since there is no data on MSI-H status directly on the GENIE database, we analyzed mutations in the mismatch-repair genes (MLH1, MSH2, MSH6, and PMS2) as surrogates for the MSI-H phenotype. To establish that alterations in such genes are surrogates for the MSI-H profile, we compared mutation count in samples with alterations in mismatch-repair genes with the whole cohort. We did not investigate the prevalence of high tumor mutational burden because this data is not readily available in the database.

To select additional targets to be included in the current analysis, we reviewed articles identified by the searches “histology-agnostic”, “tissue-agnostic”, and “basket trial cancer” in EMBASE, PubMed, and ASCO Meeting Library. Authors RCP and CSG selected targets for which prior clinical trials had demonstrated significant activity in more than one histology or for which upcoming drugs are under investigation in histology-agnostic basket trials. Selected targets include (1) mutations: KRAS G12C (22–24), KRAS G12D (25), POLE (26), POLD1 (26), BRCA1 (27), and BRCA2 (27); (2) fusions: ALK (28), ROS1 (28), NRG1 (29), FGFR (30), and (3) amplifications: HER-2 (31–33), and PD-L1 (CD274) (34). Although there is not yet clinical data with KRAS G12D inhibitors, we included this target in our analysis since it occurs in multiple tumor types (35), inhibitors are under development (25), and there is proof of concept that KRAS mutations can be actionable across histologies (23, 24).

Results are presented for each cohort identified in the GENIE database, namely: (1) STS, (2) GIST, (3) bone sarcoma, (4) uterine sarcoma, and (5) breast sarcoma. Patients included in more than one category (overlapping patients) were analyzed individually and categorized in the cohort of interest, as adjudicated by authors RCP and CSG. Patients with benign neoplasms, as designated by the WHO 2020 classification, were excluded from the analysis. We only report mutations characterized as oncogenic or likely oncogenic by OncoKB classification. Alterations of unknown significance were excluded.

## 4 Results

### 4.1 Overall patient population

We identified 7,512 samples of 6,955 patients with sarcoma in the AAACR GENIE database v12.0-Public. Patients were included in the soft tissue cohort (n=3,974), followed by GIST (n= 1,285), bone sarcoma (n= 978), uterine sarcoma (n=626) and breast sarcoma (n=99). **Table 1** contains the number of patients, samples, and specific genomic information available for each cohort. **Supplementary Table 1** contains information on patients that overlapped between groups and their final classification. Seven patients were included in more than one cohort due to the presence of two primary tumors.

**Table 1:** Number of patients, samples, and genomic information for each cohort.

	Patients (n)	Samples (n)	Genomic information; samples, n(%)
STS	3,974	4,232	Fusions: 4,232 (100%)
			Somatic mutations: 4,232 (100%)
			Copy-number alterations: 3,583 (84%)
GIST	1,285	1,447	Fusions: 1,447 (100%)
			Somatic mutations: 1,447 (100%)
			Copy-number alterations: 972 (67%)
Bone sarcoma	978	1,088	Fusions: 1,088 (100%)
			Somatic mutations: 1,088 (100%)
			Copy-number alterations: 801 (73%)
Uterine sarcoma	626	645	Fusions: 645 (100%)
			Somatic mutations: 645 (100%)
			Copy-number alterations: 528 (81%)
Breast sarcoma	99	100	Fusions: 100 (100%)
			Somatic mutations: 100 (100%)
			Copy-number alterations: 85 (85%)

#### 4.1.1 STS cohort

**Figure 1:**
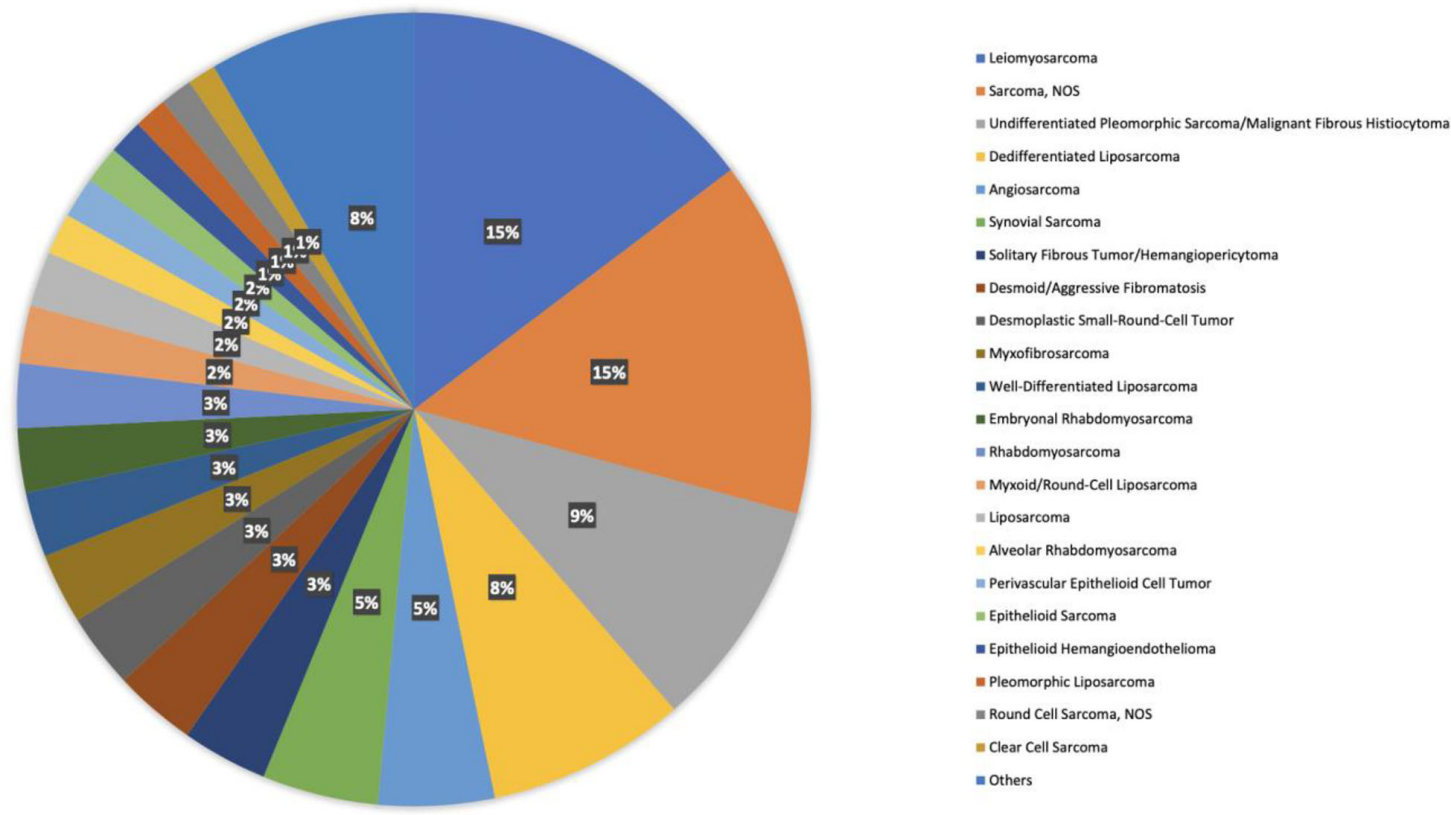
Histology distribution within the STS cohort, including all histologies representing at least 1% of patients.

There are 42 different histologies represented within the STS cohort. Most represented histologies are leiomyosarcoma (14%), sarcoma not otherwise specified (NOS) (14%), undifferentiated pleomorphic sarcoma (9%), dedifferentiated liposarcoma (8%), and angiosarcoma (4%). **Figure 1** shows histology distribution within the STS cohort, including all histologies representing at least 1% of patients. **Supplementary Table 2** provides a complete description of all histologies and the number of patients per histotype. There was a slight female predominance (51%); most samples were identified from Memorial Sloan Kettering Cancer Center (MSKCC) (51%), followed by Dana Farber Cancer Institute (DFCI) (25%), and the University of California San Francisco (UCSF) (6%). Samples were most commonly collected from primary tumors (62%), whereas 31% were from metastasis, 4% were from local recurrent tumors, and the remaining 3% were not specified.

#### 4.1.2 GIST cohort

In the GIST cohort, there was a slight male predominance (55%), and most samples were collected from the primary tumor (66%); an additional 28% of samples were collected from metastatic sites, and 6% were not specified. Regarding mutational profile, 70% of GIST samples harbored a KIT mutation, and 10% harbored a PDGFRA mutation. The remaining 20% were, therefore, classified as “wild-type”. Most samples were identified from MSKCC (41%), followed by DCFI (22%), and Johns Hopkins (JHU) (9%).

#### 4.1.3 Bone sarcoma cohort

**Figure 2:**
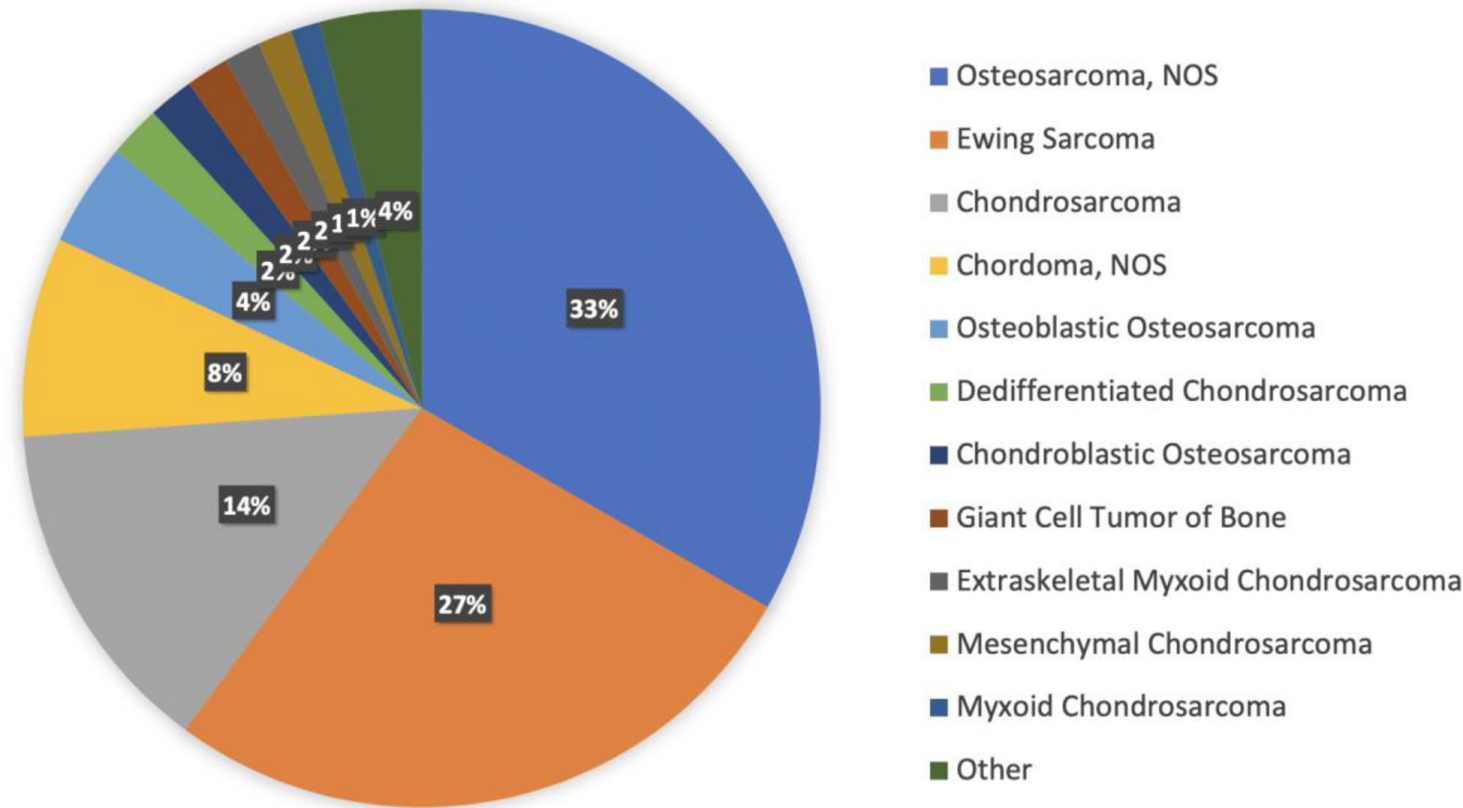
Histology distribution within the bone sarcoma cohort, including all histologies representing at least 1% of patients.

There are 22 different histologies represented within the bone sarcoma cohort. Most represented histologies are osteosarcoma NOS (33%), Ewing sarcoma (27%), chondrosarcoma NOS (14%), chordoma NOS (8%), and osteoblastic osteosarcoma (4%). **Figure 2** shows histology distribution within the bone sarcoma cohort, including all histologies representing at least 1% of patients. **Supplementary Table 3** provides a complete description of all histologies and the number of patients per histotype. There was a slight male predominance (57%); most samples were identified from MSKCC (53%), followed by DCFI (15%) and UCSF (9%). Samples were most commonly collected from primary tumors (59%), whereas 33% were from metastasis, 4% were from locally recurrent tumors, and the remaining 4% were not specified.

#### 4.1.4 Uterine sarcoma cohort

**Figure 3:**
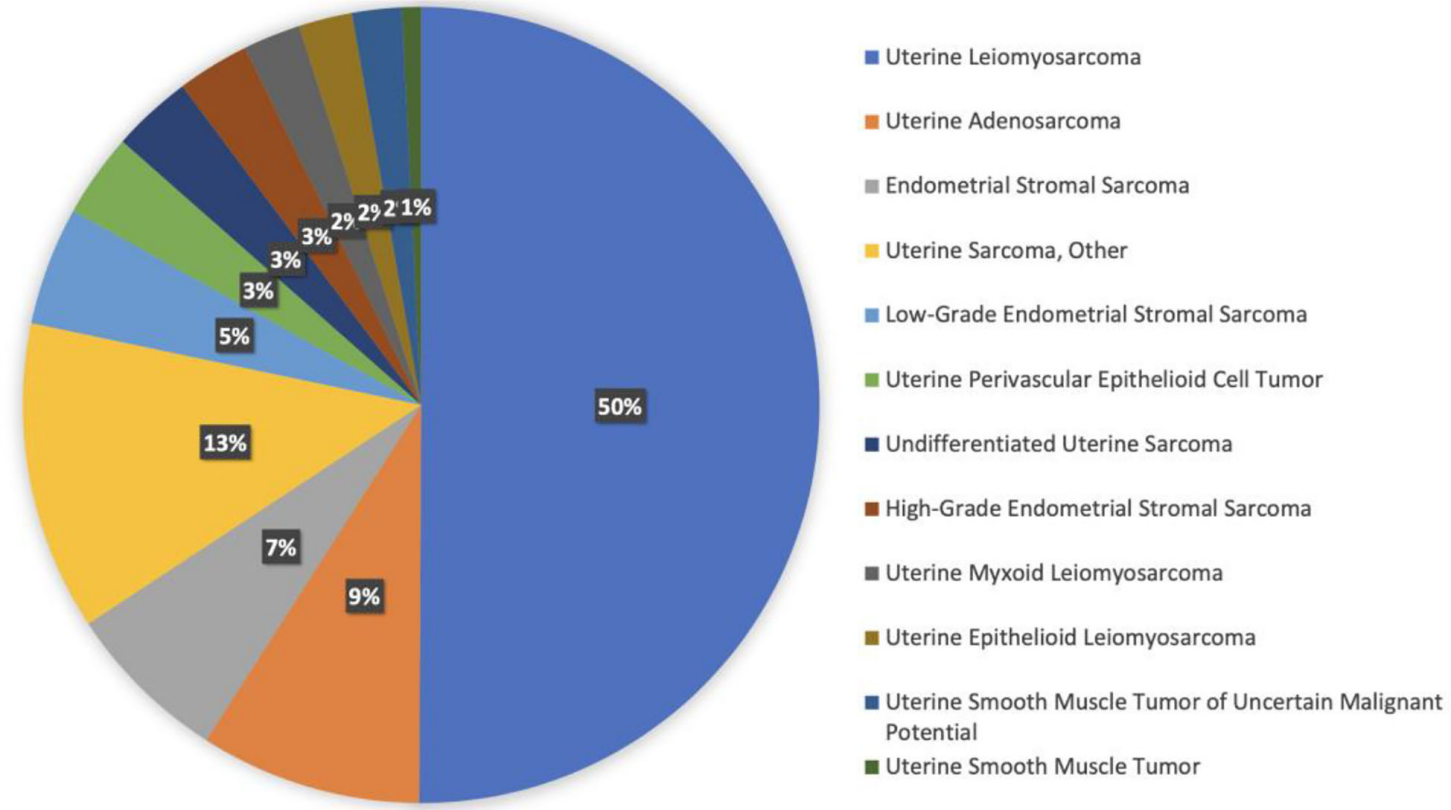
Histology distribution within the uterine sarcoma cohort.

There are 12 different histologies represented within the uterine sarcoma cohort. Most represented histologies are uterine leiomyosarcoma (49%), uterine sarcoma NOS (13%), uterine adenosarcoma (9%), endometrial stromal sarcoma NOS (6%), and low-grade endometrial stromal sarcoma (5%). **Figure 3** shows histology distribution within the uterine sarcoma cohort, including all histologies. All patients were females; most samples were identified from MSKCC (51%), followed by DFCI (25%), and UCSF (6%). Samples were most commonly collected from primary tumors (51%), whereas 45% of samples were from metastasis, and the remaining 4% were not specified.

#### 4.1.5 Breast sarcoma cohort

**Figure 4:**
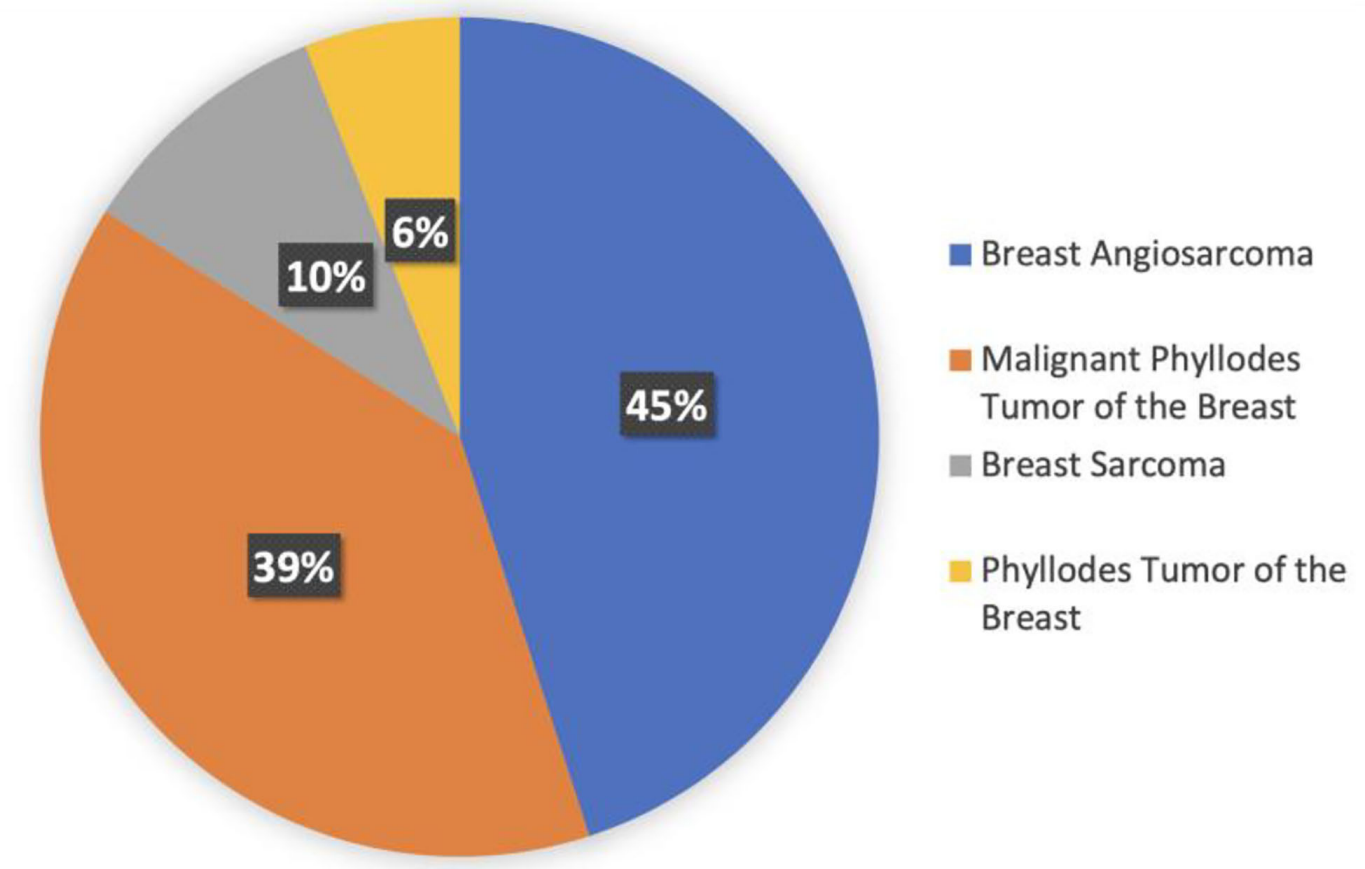
Histology distribution within the breast sarcoma cohort.

There are four different histologies represented within the breast sarcoma cohort. Most represented histologies are breast angiosarcoma (45%), malignant phyllodes tumor (39%), breast sarcoma NOS (10%), and phyllodes tumor of the breast NOS (6%). **Figure 4** shows histology distribution within the breast sarcoma cohort. All patients were female; most samples were identified from MSKCC (69%), followed by DFCI (10%) and UCSF (6%). Samples were most commonly collected from primary tumors (75%), whereas 23% of samples were from metastasis, 1% were from locally recurrent tumors, and the remaining 1% were not specified.

### 4.2 FDA-approved histology-agnostic targets

Overall, molecular alterations that could lead to an FDA-approved (except for high tumor mutational burden) were present in 148 patients (2.1%). We provide a detailed account of such cases below, and the data are summarized in **Table 2**.

**Table 2:** Approved histology-agnostic targets within each sarcoma cohort in the AACR GENIE v12.0-Public.

	NTRK fusion	Mismatch-repair gene alterations	BRAF V600E mutations	RET fusions	Total w/FDA-approved agnostic biomarker
STS (n=3,974)	28 (0.7%)	60 (1.5%)	11 (0.3%)	3 (0.1%)	102 (2.6%)
GIST (n=1,285)	1 (0.1%)	9 (0.7%)	5 (0.5%)	0	15 (1.3%)
Bone sarcoma (n=978)	4 (0.4%)	9 (0.9%)	0	1 (0.1%)	14 (1.4%)
Uterine sarcoma (n=626)	4 (0.6%)	11 (1.8%)	1 (0.2%)	1 (0.2%)	17 (2.7%)
Breast sarcoma (n=99)	0	0	0	0	0

#### 4.2.1 NTRK fusion

NTRK fusions were identified in 37 patients overall (0.5%) – 28 patients in the STS cohort (0.7%), four patients in the bone sarcoma cohort (0.4%), four patients in the uterine sarcoma cohort (0.6%), and one patient in the GIST cohort (0.1%). Fusions more commonly involved the NTRK1 gene (n=25), followed by NTRK3 (n=8) and NTRK2 (n=4) genes.

Among 28 patients harboring NTRK fusions in the STS cohort, 60% were female, and 58% had molecular profiling performed on the primary tumor specimen. Ten histologic subtypes were represented among NTRK fusion-positive STS; most common histology was sarcoma NOS (n=15), followed by fibrosarcoma (n=4), dedifferentiated liposarcoma (n=3), undifferentiated pleomorphic sarcoma (n=3), inflammatory myofibroblastic tumor (n=2), leiomyosarcoma (n=2), well-differentiated liposarcoma (n=2), angiosarcoma (n=1), liposarcoma NOS (n=1) and round cell sarcoma NOS (n=1). Interestingly, two cases of dedifferentiated liposarcoma and two cases of well-differentiated liposarcoma harbored the characteristic MDM2 amplification as a co-occurrent molecular alteration.

In the bone sarcoma cohort, among four patients harboring NTRK fusions, 75% were male, and 50% had molecular profiling performed on the primary tumor. Two histologic subtypes were represented: osteosarcoma (n=3), and extraskeletal myxoid chondrosarcoma (n=1).

In the uterine sarcoma cohort, among the four patients identified with NTRK fusions, 75% had genomic profiling in the primary tumor. Three histologic subtypes were represented: uterine sarcoma NOS (n=2), uterine adenosarcoma (n=1), and uterine leiomyosarcoma (n=1).

One patient with GIST was identified with an NTRK3 fusion. Interestingly, this patient also harbored a KIT exon 11 V560E missense mutation.

#### 4.2.2 Alterations in mismatch repair genes

Genomic alterations classified as oncogenic/likely oncogenic in MLH1, MSH2, MSH6, and PMS2 were present in 89 patients overall (1.3%), including 60 in the STS cohort (1.5%), 11 in the uterine sarcoma cohort (1.8%), nine in the bone sarcoma cohort (0.9%), and nine in the GIST cohort (0.7%).

**Figure 5:**
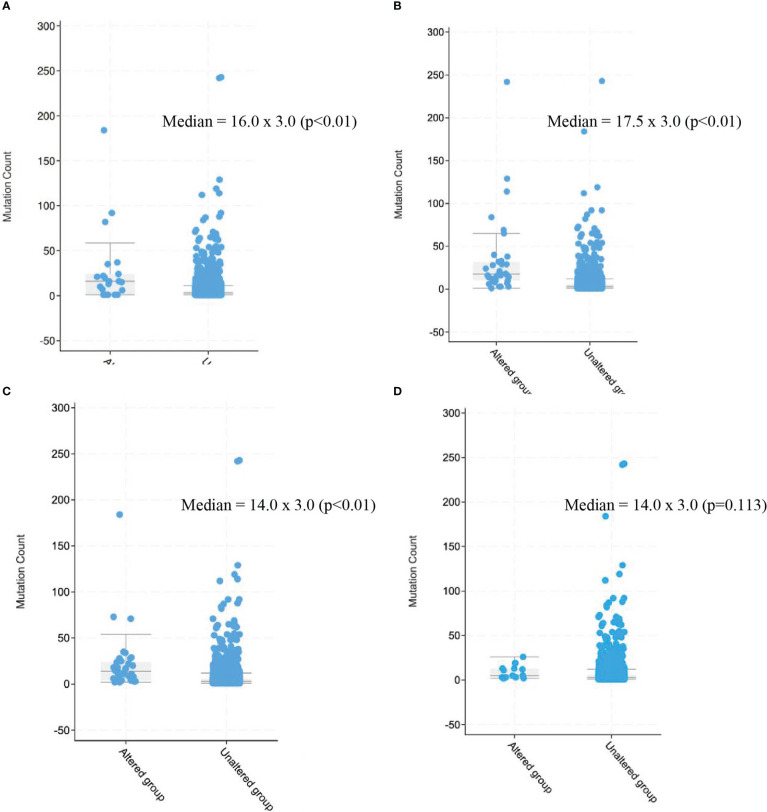
Mutation count in patients with sarcomas harboring alterations in mismatch repair genes as compared with those without alterations. **(A)** MLH1, **(B)** MSH2, **(C)** MSH6, **(D)** PMS2. 2.3 BRAF V600E mutation, BRAF V600E mutation was present in 17 patients overall (0.3%), including 11 patients in.

MLH1 alterations were present in 15 patients overall, most commonly in the STS cohort (n=13), including four patients with undifferentiated pleomorphic sarcoma, three patients with sarcoma NOS, two patients with rhabdomyosarcoma, two patients with intimal sarcoma, one patient with leiomyosarcoma, and one patient with a radiation-associated sarcoma. MLH1 alterations were also seen in one patient in the GIST cohort and one patient in the bone sarcoma cohort (osteosarcoma). Most common alterations were truncating (n=6), followed by splice-site mutations (n=5), structural variations (SV)/fusions (n=4) and missense mutations (n=1). MLH1-altered sarcomas had a higher mutation count than sarcomas without MLH1 alteration (median 16.0 x 3.0, p<0.01) (**Figure 5A**).

MSH2 alterations were present in 32 patients overall, most commonly in the STS cohort (n=22), including nine patients with sarcoma NOS, three patients with undifferentiated pleomorphic sarcoma, two patients with leiomyosarcoma, two patients with rhabdomyosarcoma, one patient with pleomorphic liposarcoma, one patient with well-differentiated liposarcoma, one patient with liposarcoma NOS, one patient with solitary fibrous tumor, one patient with angiosarcoma, and one patient with alveolar soft part sarcoma. MSH2 alterations were also seen in four patients in the GIST cohort, four in the uterine sarcoma cohort – three leiomyosarcomas and one adenosarcoma – and two in the bone sarcoma cohort (conventional type chordoma and chondrosarcoma). Most common alterations were splice-site mutations (n=15), followed by truncating (n=12), SV/fusions (n=3) and missense mutations (n=2). MSH2-altered sarcomas had a higher mutation count than sarcomas without MSH2 alteration (median 17.5 x 3.0, p<0.01) (**Figure 5B**).

MSH6 alterations were present in 34 patients overall, most commonly in the STS cohort (n=21), including six patients with leiomyosarcoma, five patients with undifferentiated pleomorphic sarcoma, five patients with sarcoma NOS, two patients with pleomorphic rhabdomyosarcoma, one patient angiosarcoma, one patient with dedifferentiated liposarcoma, and one with follicular dendritic cell sarcoma. MSH6 alterations were also seen in six patients in the uterine sarcoma cohort – two leiomyosarcoma, two sarcoma NOS, one undifferentiated uterine sarcoma, one low-grade endometrial stromal sarcoma –, five patients in the bone sarcoma cohort – osteosarcoma (n=4), Ewing sarcoma (n=1) –, and two patients in the GIST cohort. Two patients had alterations in both MLH1 and MSH6 (both undifferentiated pleomorphic sarcoma). Most common alterations were truncating (n-30), followed by splice-site mutations (n=5), SV/fusions (n=4) and missense mutations (n=1). MSH6-altered sarcomas had a higher mutation count than sarcomas without MSH6 alteration (median 14.0 x 3.0, p<0.01) (**Figure 5C**).

PMS2 alterations were present in 10 patients overall, most commonly in the STS cohort (n=6), including two patients with leiomyosarcoma, two patients with sarcoma NOS, and one patient each with myxofibrosarcoma and pleomorphic liposarcoma. PMS2 alterations were also seen in two patients with GIST, one patient in the bone sarcoma cohort (giant cell tumor of bone), and one patient in the uterine sarcoma cohort (leiomyosarcoma). Most common alterations were truncating (n=5), followed by splice-site mutations (n=3), SV/fusions (n=2) and missense mutations (n=2). PMS2-altered sarcomas did not demonstrate higher mutation count than sarcomas without PMS2 alteration (median 14.0 x 3.0, p=0.113) (**figure 5D**).

#### 4.2.3 BRAF V600E mutation

BRAF V600E mutation was present in 17 patients overall (0.3%), including 11 patients in the STS cohort (0.3%), five patients in the GIST cohort (0.5%), and one in the uterine sarcoma cohort (0.2%). In the STS cohort, BRAF V600E mutations were identified in patients diagnosed with sarcoma NOS (n=4), angiosarcoma (n=2), solitary fibrous tumor (n=1), desmoid fibromatosis (n=1), round cell sarcoma NOS (n=1), embryonal rhabdomyosarcoma (n=1), and undifferentiated pleomorphic sarcoma (n=1). All patients with BRAF V600E mutated GIST were wild-type for KIT and PDGFRA. The one patient with BRAF V600E mutation in the uterine sarcoma cohort was diagnosed with uterine leiomyosarcoma.

#### 4.2.4 RET fusions

RET fusions were identified in three patients in the STS cohort – sarcoma NOS (n=2) and undifferentiated pleomorphic sarcoma (n=1) –, one patient in the bone sarcoma cohort (osteosarcoma), and one patient in the uterine sarcoma cohort (leiomyosarcoma).

### 4.3 Other potential histology-agnostic targets

Overall, molecular alterations that are under investigation as potential histology-agnostic predictive biomarkers in the future were present in 202 patients (2.9%). We provide a detailed account of such cases below, and the data are summarized in **Tables 3**–**5**.

**Table 3:** Prevalence of gene mutations being investigated as potential histology-agnostic predictive biomarkers in sarcoma samples in the AACR GENIE v12.0-Public.

	KRAS G12C	KRAS G12D	POLE/POLD1	BRCA1	BRCA2	Total
STS (n=3,974)	6 (0.2%)	16 (0.4%)	0	23 (0.6%)	25 (0.6%)	70 (1.8%)
GIST (n=1,285)	0	1 (0.1%)	0	1 (0.1%)	1 (0.1%)	3 (0.3%)
Bone sarcoma (n=978)	0	1 (0.1%)	0	4 (0.4%)	6 (0.6%)	11 (1.1%)
Uterine sarcoma (n=626)	1 (0.2%)	4 (0.6%)	0	1 (0.2%)	10 (1.6%)	16 (2.6%)
Breast sarcoma (n=99)	1 (1%)	1 (1%)	0	0	0	2 (2%)

**Table 4:** Prevalence of gene fusions being investigated as potential histology-agnostic predictive biomarkers in sarcoma samples in the AACR GENIE database v12.0-Public.

	NRG1	ALK	ROS1	FGFR	Total
Soft-tissue sarcoma (n=3,974)	2 (0.1%)	23 (0.6%)	14 (0.4%)	16 (0.4%)	55 (1.4%)
GIST (n=1,285)	0	1 (0.1%)	0	1 (0.1%)	2 (0.2%)
Bone sarcoma (n=978)	1 (0.1%)	0	1 (0.1%)	3 (0.3%)	5 (0.5%)
Uterine sarcoma (n=626)	0	6 (1%)	0	2 (0.3%)	8 (1.3%)
Breast sarcoma (n=99)	0	0	0	0	0

**Table 5:** Prevalence of gene amplifications being investigated as potential histology-agnostic predictive biomarkers in sarcoma samples in the AACR GENIE v12.0-Public.

	HER-2	CD274	Total
Soft-tissue sarcoma (n=3,974)	3 (0.1%)	17 (0.4%)	20 (0.5%)
GIST (n=1,285)	0	0	0
Bone sarcoma (n=978)	1 (0.1%)	4 (0.4%)	5 (0.5%)
Uterine sarcoma (n=626)	0	0	0
Breast sarcoma (n=99)	0	0	0

#### 4.3.1 Potentially histology-agnostic actionable mutations

KRAS G12C mutation was present in eight patients overall – six patients in the STS cohort, one in the uterine sarcoma cohort (undifferentiated uterine sarcoma), and one in the breast sarcoma cohort (angiosarcoma). In the STS cohort, KRAS G12C mutations were identified angiosarcoma (n=2), sarcoma NOS (n=2), myxoid/round-cell liposarcoma (n=1), and undifferentiated pleomorphic sarcoma (n=1).

KRAS G12D mutation was present in 23 patients overall – 16 patients in the STS cohort, four patients in the uterine sarcoma cohort, one patient in the bone sarcoma cohort (Ewing sarcoma), one patient in the breast sarcoma cohort (malignant phyllodes tumor), and one patient in the GIST cohort. In the STS cohort, these mutations were identified in patients with sarcoma NOS (n=6), rhabdomyosarcoma (n=3), histiocytic dendritic cell sarcoma (n=2), and one each of inflammatory myofibroblastic tumor, leiomyosarcoma, synovial sarcoma, undifferentiated pleomorphic sarcoma, dedifferentiated liposarcoma.

BRCA1 mutations were identified in 29 patients – 23 in the STS cohort, four with bone cancer, one with GIST, and one with uterine sarcoma (uterine leiomyosarcoma). Within the STS cohort, oncogenic BRCA1 mutations were identified in patients diagnosed with leiomyosarcoma (n=8), myxofibrosarcoma (n=3), undifferentiated pleomorphic sarcoma (n=3), sarcoma NOS (n=2), inflammatory myofibroblastic tumor (n=1), Ewing sarcoma of soft tissue (n=1), pleomorphic liposarcoma (n=1), solitary fibrous tumor (n=1), synovial sarcoma (n=1), dedifferentiated liposarcoma (n=1), and spindle cell rhabdomyosarcoma (n=1). In the bone sarcoma cohort, such mutations were seen in patients with osteosarcoma (n=3) and Ewing sarcoma (n=1).

BRCA2 mutations were identified in 42 patients – 25 in the STS cohort, 10 with uterine sarcoma, six with bone sarcoma, and one with GIST. Within the STS cohort, oncogenic BRCA2 mutations were identified in patients diagnosed with leiomyosarcoma (n=7), sarcoma NOS (n=6), angiosarcoma (n=3), epithelioid sarcoma (n=2), embryonal rhabdomyosarcoma (n=2), radiation-associated sarcoma (n=1), round cell sarcoma NOS (n=1), solitary fibrous tumor (n=1), undifferentiated pleomorphic sarcoma (n=1), and dedifferentiated liposarcoma (n=1). In the uterine sarcoma cohort, these mutations were identified in uterine leiomyosarcoma (n=7), uterine sarcoma NOS (n=2), and undifferentiated uterine sarcoma (n=1). In the bone sarcoma cohort, such mutations were seen in patients with osteosarcoma (n=4), chordoma (n=1), and chondrosarcoma (n=1).

No cases were identified with OncoKB oncogenic/likely oncogenic POLE or POLD1 mutations.

#### 4.3.2 Potentially histology-agnostic actionable fusions

NRG1 fusions were present in two patients in the STS cohort (dedifferentiated liposarcoma and synovial sarcoma) and one in the bone sarcoma cohort (osteoblastic osteosarcoma).

ALK fusions were present in 23 patients in the STS cohort – inflammatory myofibroblastic tumor (n=16), leiomyosarcoma (n=2), rhabdomyosarcoma (n=2), sarcoma NOS (n=2), and myxofibrosarcoma (n=1) –, six patients in uterine sarcoma – uterine leiomyosarcoma (n=2), uterine adenosarcoma (n=1), uterine sarcoma NOS (n=1), and uterine smooth muscle tumor of uncertain malignant potential (n=1) –, and one patient in the GIST cohort.

ROS1 fusions were seen in 14 patients in the STS cohort – dedifferentiated liposarcoma (n=4), inflammatory myofibroblastic tumor (n=4), leiomyosarcoma (n=1), liposarcoma (n=1), myxoid/round cell liposarcoma (n=1), PEComa (n=1), synovial sarcoma (n=1), undifferentiated pleomorphic sarcoma (n=1) –, and one patient in the bone sarcoma cohort (osteosarcoma).

FGFR rearrangements were seen in 16 patients in the STS cohort – dedifferentiated liposarcoma (n=4), sarcoma NOS (n=4), undifferentiated pleomorphic sarcoma (n=3), myxofibrosarcoma (n=1), pleomorphic liposarcoma (n=1), rhabdomyosarcoma (n=1), synovial sarcoma a(n=1), and well-differentiated liposarcoma (n=1) –, three patients in the bone sarcoma cohort – osteosarcoma (n=2), and Ewing sarcoma (n=1) –, two patients in the uterine sarcoma cohort (uterine leiomyosarcoma and uterine sarcoma NOS) –, and one patient with GIST.

#### 4.3.3 Potentially histology-agnostic actionable amplifications

HER-2 amplification was seen in four patients: three in the STS cohort – leiomyosarcoma, sarcoma NOS and synovial sarcoma – and one in the bone sarcoma cohort (osteosarcoma).

CD274 (PD-L1) amplification was identified in 21 patients, including 17 patients in the STS cohort – dedifferentiated liposarcoma (n=8), undifferentiated pleomorphic sarcoma (n=5), sarcoma NOS (n=2), myxofibrosarcoma (n=1), pleomorphic liposarcoma (n=1) –, and four patients in the bone sarcoma cohort (all osteosarcoma).

## 5 Discussion

By exploring a public large-scale genomic database, we identified that 2.1% of patients with sarcoma could be eligible for current histology-agnostic approved drugs and that an additional 2.9% could be eligible for future potential histology-agnostic indications. Interestingly, these actionable alterations were present in a wide variety of histologies in both soft-tissue and, yet to a lesser degree, bone sarcomas.

Although sarcoma is a heterogeneous group of malignancies with subtype-specific prognosis and biologic features, a one-size-fits-all approach to therapy has dominated systemic therapies for soft tissue and bone sarcomas in the past decades. However, more recently, isolated successes of targeted therapies have led to renewed interest in biomarker- and histology-specific development of novel agents. Strikingly, multiple retrospective analyses indicate the potential of next-generation sequencing to identify actionable alterations in tumors from patients with advanced bone and soft tissue sarcoma. For example, in an interesting analysis of 102 consecutive sarcoma patients treated at MD Anderson Cancer Center, 61% were identified to carry a potentially actionable molecular alteration; encouragingly, 16% eventually received personalized therapy, with 50% achieving clinical benefit (36). Accordingly, a report from the Moffitt Cancer Center demonstrated that tumors from 49% of 114 patients diagnosed with sarcoma had a molecular alteration deemed as actionable; 15 of these patients were treated with drugs guided by molecular results, and 26% achieved a clinical benefit from targeted therapy (37).

Moreover, a growing body of evidence suggests the value of molecularly targeted therapies to improve outcomes for patients with sarcoma. A recent trial evaluating the role of comprehensive molecular profiling in rare cancers suggests that integration of genomic and transcriptomic analysis in clinical practice can lead to a specific management strategy, including diagnostic reevaluation, genetic counseling, and experimental treatment, beyond current guidelines, in 88% of the cases (38). Of note, analyzing specifically the patients with soft tissue sarcoma within this trial that were eventually treated with molecularly informed therapy, 35% achieved a progression-free survival benefit of at least 30% longer to targeted therapy compared to prior treatment. In addition, a recent publication analyzing data from two French centers demonstrated the benefit of molecularly targeted agents in 214 patients enrolled in early-phase trials (39).

However, despite the data provided above, the development of specific targeted therapies for sarcomas is challenging due to the rarity of such neoplasms and difficulties with the recruitment of patients and with funding for studies of new drugs in sarcomas. Therefore, the modern regulatory pathway of histology-agnostic approvals is of particular interest for patients with sarcoma, as it can provide patients with standard-of-care targeted/immunotherapy agents with the potential to modify the natural history of metastatic disease. For illustration, provided there is no direct comparison between current standard of care chemotherapy and NTRK-targeted agents for patients with sarcoma, an analysis presented at the ASCO Annual Meeting 2021 supports the superiority of targeted therapy in this context. In this intra-patient comparison (n=149), the median time to progression on prior therapy was 3.0 months, while progression-free survival for larotrectinib was 33.0 months. Moreover, the authors identified that 74% of patients had a growth modulation index of at least 1.33, meaning that these patients had a benefit at least 33% longer for larotrectinib compared to prior therapy (40).

On the other hand, it is important to recognize that it is expected that for most histology-agnostic approvals, very few patients with sarcoma will be enrolled in the trial leading for approval. With the exception of trials leading to approval of NTRK fusions, in which soft tissue sarcoma was the predominant tumor type enrolled (21% - 26% of all patients), there is limited information on the activity of currently approved histology agnostic drugs in sarcoma for other approvals (20). For illustration, in the largest trial among those leading to approval of pembrolizumab for MSI-H solid tumors, only 4% of patients were diagnosed with sarcoma. Moreover, in the trials leading to the approval of pembrolizumab for TMB-H tumors and dabrafenib and trametinib for BRAFV600E mutated tumors, no patients with sarcoma were enrolled. In this sense, post-approval real-world data will be essential to evaluate the specific activity of these agents in sarcoma. It would be of interest for national and international sarcoma societies to encourage such analysis.

One question that can arise in clinical practice following this analysis is about which patients with advanced sarcoma should have broad somatic next-generation sequencing of their tumors. While this is not well established to date, and there is not a single answer suitable for different socioeconomic paradigms, we argue that, in the ideal situation, all patients with advanced sarcoma should undergo such testing. The reasoning for this is not only the potential for diagnostic changes with next-generation sequencing but also the identification of histology-specific and histology-agnostic targets for standard-of-care and clinical trial options. We believe that future discussions should focus on how to enable access to testing for all patients diagnosed with sarcoma rather than concentrating on whether testing should be performed.

There are several limitations to the current analysis. First, there is no data on treatment outcomes and efficacy of targeted therapies for the alterations identified. Therefore, the true actionability of the described molecular alterations in sarcomas remains to be elucidated. The exact number of patients for whom outcomes would be impacted based on the genomic results is unknown. Second, there are limited data regarding the pathologic diagnosis within the AACR GENIE database. Although most cases were referred from tertiary reference sarcoma centers, there is potential for diagnostic uncertainties, and molecular profiling might not have been specifically performed for diagnostic confirmation in most cases. Moreover, there is an inherent bias associated with patient selection from institutions that participate in the AACR GENIE database, with access to comprehensive tumor sequencing testing that at the time is not yet standard of care for patients with sarcoma.

## 6 Conclusions

By analyzing a public large-scale genomic database, we identify that 5% of patients with advanced sarcoma are eligible for current histology-agnostic approved drugs or future potential agnostic indications. Our data suggest that somatic next-generation sequencing can be considered in clinical practice to guide standard-of-care treatments and clinical trial options for patients with soft tissue and bone sarcoma.

## Data availability statement

The original contributions presented in the study are included in the article/**Supplementary Material**. Further inquiries can be directed to the corresponding author.

## Author contributions

RP and CS-G both contributted to design, analysis, and writing of final manuscript. RP was responsible for writing the first draft of the manuscript. Both authors contributed to the article and approved the submitted version.
