# Supplementary material for: Analysis of histology-agnostic targets among soft tissue and bone sarcomas in the AACR GENIE database
Source: Front Oncol. 2023 Jan 18;12:1079909. doi: 10.3389/fonc.2022.1079909 (PMC9890057; doi:10.3389/fonc.2022.1079909)
Supplement: Supplementary file 1 [file Table_1.docx]

**Supplementary material:**

**Supplementary table 1.** Overlapping patient characteristics and final adjudication

|  | Overlap | Final classification | Number of Samples |
| --- | --- | --- | --- |
| GENIE-DFCI-171922 | Breast sarcoma and STS | Breast Sarcoma | 2 |
| GENIE-MSK-P-0007324 | Breast sarcoma and STS | Breast Sarcoma | 2 |
| GENIE-MSK-P-0027469 | Breast sarcoma and STS | Breast Sarcoma | 2 |
| GENIE-DFCI-000301 | Uterine sarcoma and STS | Uterine Sarcoma | 2 |
| GENIE-DFCI-001231 | Uterine sarcoma and STS | Uterine Sarcoma | 2 |
| GENIE-DFCI-0947722 | Uterine sarcoma and STS | Uterine Sarcoma | 2 |
| GENIE-DFCI-204291 | Uterine sarcoma and STS | Uterine Sarcoma | 2 |
| GENIE-MSK-P-0019715 | Uterine sarcoma and STS | Uterine Sarcoma | 4 |
| GENIE-MSK-P-0033927 | Uterine sarcoma and STS | Uterine Sarcoma | 2 |
| GENIE-DFCI-005239 | GIST and STS | GIST | 2 |
| GENIE-MSK-P-0004657 | GIST and STS | Included in both cohorts (two primary cancers) | 2 |
| GENIE-MSK-P-0023543 | GIST and STS | Included in both cohorts (two primary cancers) | 2 |
| GENIE-MSK-P-0026733 | GIST and STS | Included in both cohorts (two primary cancers) | 2 |
| GENIE-MSK-P-0030889 | GIST and STS | Included in both cohorts (two primary cancers) | 2 |
| GENIE-MSK-P-0039199 | GIST and STS | Included in both cohorts (two primary cancers) | 2 |
| GENIE-MSK-P-0013530 | Bone sarcoma and STS | Bone sarcoma | 2 |
| GENIE-MSK-P-0023399 | Bone sarcoma and STS | Bone sarcoma | 2 |
| GENIE-MSK-P-0033015 | Bone sarcoma and STS | Included in both cohorts (two primary cancers) | 2 |
| GENIE-MSK-P-0034612 | Bone sarcoma and STS | Included in both cohorts (two primary cancers) | 2 |
| GENIE-MSK-P-0045639 | Bone sarcoma and STS | Bone sarcoma | 3 |
| GENIE-MSK-P-0056684 | Bone sarcoma and STS | Bone sarcoma | 3 |
| GENIE-UCSF-11257 | Bone sarcoma and STS | Bone sarcoma | 2 |
| GENIE-UCSF-3695 | Bone sarcoma and STS | Bone sarcoma | 2 |

**Supplementary table 2.** Histotypes in the STS cohort.

| Category | Number of samples |
| --- | --- |
| Leiomyosarcoma | 622 |
| Sarcoma, NOS | 615 |
| Undifferentiated Pleomorphic Sarcoma/Malignant Fibrous Histiocytoma/High-Grade Spindle Cell Sarcoma | 398 |
| Dedifferentiated Liposarcoma | 343 |
| Angiosarcoma | 200 |
| Synovial Sarcoma | 200 |
| Solitary Fibrous Tumor/Hemangiopericytoma | 148 |
| Desmoid/Aggressive Fibromatosis | 142 |
| Desmoplastic Small-Round-Cell Tumor | 129 |
| Myxofibrosarcoma | 122 |
| Well-Differentiated Liposarcoma | 112 |
| Embryonal Rhabdomyosarcoma | 111 |
| Rhabdomyosarcoma | 111 |
| Myxoid/Round-Cell Liposarcoma | 99 |
| Liposarcoma | 95 |
| Alveolar Rhabdomyosarcoma | 71 |
| Perivascular Epithelioid Cell Tumor | 71 |
| Epithelioid Sarcoma | 65 |
| Epithelioid Hemangioendothelioma | 60 |
| Pleomorphic Liposarcoma | 57 |
| Round Cell Sarcoma, NOS | 56 |
| Clear Cell Sarcoma | 49 |
| Inflammatory Myofibroblastic Tumor | 42 |
| Alveolar Soft Part Sarcoma | 37 |
| Intimal Sarcoma | 32 |
| Radiation-Associated Sarcoma | 31 |
| Fibrosarcoma | 27 |
| Follicular Dendritic Cell Sarcoma | 25 |
| Sclerosing Epithelioid Fibrosarcoma | 23 |
| Histiocytic Dendritic Cell Sarcoma | 21 |
| Pleomorphic Rhabdomyosarcoma | 21 |
| Spindle Cell Rhabdomyosarcoma | 21 |
| Low-Grade Fibromyxoid Sarcoma | 17 |
| Ewing Sarcoma of Soft Tissue | 15 |
| Atypical Lipomatous Tumor | 10 |
| Proximal-Type Epithelioid Sarcoma | 9 |
| Tenosynovial Giant Cell Tumor Diffuse Type | 7 |
| Spindle Cell/Sclerosing Rhabdomyosarcoma | 6 |
| Ossifying Fibromyxoid Tumor | 4 |
| Glomangiosarcoma | 3 |
| Pseudomyogenic Hemangioendothelioma | 3 |
| Interdigitating Dendritic Cell Sarcoma | 2 |

**Supplementary table 3.** Histotypes in the bone sarcoma cohort.

| Histology | Number of samples |
| --- | --- |
| Osteosarcoma, NOS | 363 |
| Ewing Sarcoma | 292 |
| Chondrosarcoma | 150 |
| Chordoma, NOS | 88 |
| Osteoblastic Osteosarcoma | 46 |
| Dedifferentiated Chondrosarcoma | 23 |
| Chondroblastic Osteosarcoma | 20 |
| Giant Cell Tumor of Bone | 19 |
| Extraskeletal Myxoid Chondrosarcoma | 16 |
| Mesenchymal Chondrosarcoma | 15 |
| Myxoid Chondrosarcoma | 13 |
| Conventional Type Chordoma | 10 |
| High-Grade Surface Osteosarcoma | 9 |
| Adamantinoma | 6 |
| Periosteal Osteosarcoma | 5 |
| Secondary Osteosarcoma | 4 |
| Parosteal Osteosarcoma | 3 |
| Fibroblastic Osteosarcoma | 2 |
| Small Cell Osteosarcoma | 2 |
| Telangiectatic Osteosarcoma | 2 |
| Dedifferentiated Chordoma | 1 |
| Low-Grade Central Osteosarcoma | 1 |
